# Effects of antihypertensives with and without IL-6 lowering properties on long-term blood pressure control: The prospective HELIUS cohort

**DOI:** 10.1016/j.ijcrp.2024.200358

**Published:** 2024-12-11

**Authors:** Hillman Batuo, Eva van der Linden, Henrike Galenkamp, Eric Moll van Charante, Bert-Jan van der Born, Felix P. Chilunga

**Affiliations:** a Department of Public and Occupational Health, Amsterdam Public health Research Institute, Amsterdam university Medical Center, University of Amsterdam, Amsterdam, the Netherlands; b Department of Vascular Medicine, Amsterdam Cardiovascular Sciences, Amsterdam UMC, University of Amsterdam, Amsterdam, the Netherlands

## Abstract

**Background:**

Chronic inflammation is a well-recognized contributor to hypertension pathogenesis. However, the role of targeting inflammation in hypertension treatment, particularly through modulation of inflammatory markers like interleukin-6 (IL-6), remains less understood. We investigated the effects of antihypertensive medications with and without IL-6-lowering properties on long-term blood pressure (BP) control in a multi-ethnic cohort in the Netherlands.

**Methods:**

Participants from HELIUS cohort receiving hypertension treatment were followed over six years. BP control at follow-up was determined using WHO criteria. Due to unavailability of IL-6 data, a literature review was conducted to classify antihypertensives based on their IL-6-lowering properties — a proxy approach. Logistic regression models were used to assess associations between the IL-6-lowering potential of antihypertensives and BP control, both within and between antihypertensive classes. Effect modification by ethnicity was explored.

**Results:**

A total of 1510 participants were included (mean age 57 years, 62 % women). Within the calcium channel blocker (CCB) class, medications with IL-6-lowering properties (amlodipine and barnidipine) were associated with superior BP control (aOR 1.41, 95 % confidence interval 1.05–1.90) compared to other CCBs (lercanidipine, nifedipine, verapamil, clevidipine, diltiazem). No significant associations were observed within angiotensin receptor blockers (ARBs) or angiotensin-converting enzyme inhibitors (ACEIs), between different antihypertensive drug classes, nor across ethnic groups.

**Conclusion:**

Amlodipine and barnidipine were associated with better BP control compared to other CCBs. Our findings provide an important starting point for understanding the role of IL-6 in hypertension management. Further studies are warranted to confirm these observations by directly measuring IL-6 levels and investigating underlying mechanisms.

## Highlights

• The benefit of using anti-hypertensive medications that reduce inflammation is unclear.
• Amlodipine and barnidipine (with IL-6-lowering properties) showed superior blood pressure control compared to other CCBs.
• No similar effects were observed with other anti-hypertensive drug classes such as ARBs or ACEIs.
• Further research is needed to directly measure IL-6 levels and investigate underlying pathways.

## 1 Introduction

Hypertension is one of the biggest global health challenges, affecting over one billion people in 2019 [1]. While lifestyle factors like unhealthy diet, physical inactivity, and weight management are included in hypertension management strategies, control rates remain far from optimal [1]. For instance, in 2019, only 47 % of those treated for hypertension globally achieved blood pressure control [1]. New strategies are clearly needed to supplement existing management approaches.

Chronic inflammation is a contributor to hypertension and could be a target for novel control strategies [2,3]. Chronic inflammation can trigger oxidative stress, impair endothelial function, and reduce nitric oxide availability, all of which contribute to blood vessel constriction [2,3]. Monoclonal antibodies like Canakinumab [4], as well as other anti-inflammatory agents like Colchicine and Methotrexate have shown promise in reducing cardiovascular events in atherosclerosis and could possibly be explored in hypertension [4]. However, treatment with monoclonal antibodies and other biologics has been associated with a significant increase in infection-related deaths due to immunosuppressive effects [4]. Therefore, novel ways to achieve inflammation reduction without immunosuppression are needed.

Even though some antihypertensives possess anti-inflammatory properties [5], they are not typically associated with immunosuppression [5]. The intrinsic anti-inflammatory properties of these drugs could be exploited to address the immunosuppressive effects of biologics and pave the way for a novel class of antihypertensives that combine antihypertensive and anti-inflammatory action. Despite this potential, no human studies have investigated the relationship between the anti-inflammatory properties of anti-hypertensives and blood pressure control.

The pleiotropic effects of Interleukin 6 (IL-6), a pro-inflammatory cytokine involved in systemic inflammation, make it an ideal target for controlling multiple inflammatory pathways from a single point [6,7]. In the liver, IL-6 triggers the production of acute-phase proteins like C-reactive protein (CRP) and fibrinogen, markers of inflammation [6,7]. On endothelial cells, it promotes the movement of monocytes and macrophages to infection sites [6,7]. For CD4^+^ T cells, it can boost the development of Th17 cells, which promote inflammation, while sometimes hindering the function of regulatory T cells (Tregs) that keep the immune response in check [6,7]. B cells, responsible for antibody production, are also stimulated by IL-6 [6,7]. Furthermore, it can influence the production of other cytokines like TNF-alpha and IL-1 [6,7].

Considering this central role of IL-6 in inflammation, and inflammation in hypertension pathogenesis, we investigated associations between use of antihypertensives with and without anti-IL-6 properties and long-term blood pressure control in a multi-ethnic cohort in the Netherlands (Dutch, Moroccan, Ghanaian, South-Asian Surinamese, African Surinamese and Turkish origin). We hypothesized that antihypertensives with IL-6 lowering properties would be superior at controlling blood pressure in the general population compared to those without IL-6 lowering properties. We further hypothesized that this superiority would be more pronounced in populations with higher levels of chronic inflammation (e.g., ethnic minority populations in Europe) compared to those with lower levels (e.g., European descendant population) [8].

## 2 Methodology

### 2.1 Study populations and study design

The study was conducted within the population-based, prospective Healthy Life In an Urban Setting (HELIUS) cohort [9]. This cohort aims to gain insights into the causes of the unequal burden of disease across ethnic populations and ultimately enable improvement in healthcare, prevention strategies, and health outcomes in Amsterdam, the Netherlands [9]. A full description of the cohort is provided in detail elsewhere [9]. In brief, HELIUS recruited 24,780 multi-ethnic participants of Dutch, Ghanaian, South-Asian Surinamese, African Surinamese, Moroccan, and Turkish origin between 2011 and 2015. Participants were randomly selected, stratified by ethnicity, from the municipal registry. Out of these, 22,162 completed a questionnaire and underwent a physical examination. From this group, 11,035 participants were included in followed up between 2019 and 2022 (mean follow-up of 6.4 years). Ethnicity was determined based on the country of birth of the participant as well as their parents, following Statistics Netherlands criteria [10]. The detailed ethnicity criteria are provided in Appendix 1 (Supplementary Material). Participants completed structured questionnaires, physical examination, and biological material collected (e.g. blood, urine) at baseline and follow-up.

**Fig. 1:**
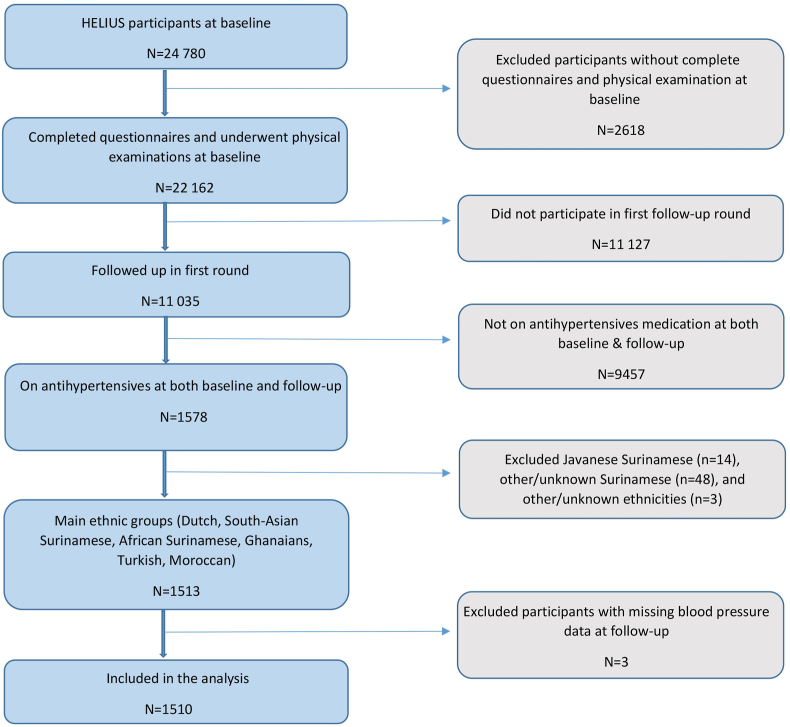
Flow chart of participation.

For this study, we selected 1578 participants who had hypertension at baseline and were consistently treated with antihypertensives throughout the study period. We then analyzed their blood pressure at follow-up. Sixty-five individuals were excluded due to ethnicities outside the study's scope (14 Javanese, 48 other Surinamese, and 3 unknown), and 3 were excluded for missing blood pressure data at follow-up. Ultimately, 1510 participants were included in the final analysis (Fig. 1).

### Ethical approval

Ethical approval, in accordance with the ethical standards as laid down in the 1964 Declaration of Helsinki and its later amendments, for the HELIUS study was obtained from the Medical Ethical Committee of the Academic Medical Center. The Approval number/code is: 10/100, NL32251.018.10. All participants provided written informed consent prior to enrollment in the study.

### 2.2 Baseline measurements

#### 2.2.1 Hypertension and anti-hypertensives

Blood pressure at baseline was assessed in a seated position using a Microlife WatchBP machine in mmHg. The mean of two measurements was used. Hypertension was defined according to WHO criteria: systolic BP < 140 mmHg and diastolic BP < 90 mmHg, and use of antihypertensive medications. Only participants with hypertension and taking antihypertensive medications were included. Participants were instructed to bring all prescribed medications to the research site. The use of antihypertensive medication was confirmed by examining the medications brought. The antihypertensives were then classified according to the Anatomical Therapeutic Chemical (ATC) classification system to verify the type of medication used. A total of 56 unique antihypertensive medications were identified in the study population (Appendix 2; Supplementary Material).

Since the HELIUS cohort lacks direct IL-6 data, we conducted a comprehensive literature review to evaluate the IL-6-lowering effects of the 56 antihypertensive medications as a proxy approach. IL-6 is one of the most extensively studied cytokines [11], and the antihypertensive medications included in our analysis are among the most commonly prescribed [12], ensuring that there was adequate literature available to evaluate their IL-6-modulating properties. Moreover, we incorporated both positive and null findings from the literature, which minimized bias from focusing solely on studies reporting an effect and provides a balanced foundation for this analysis. Despite its limitations, the proxy approach was to providing preliminary insights, which can be built upon with more detailed and better study designs.

Prominent databases like PubMed, EMBASE, SCOPUS, Web of Science and MEDLINE were searched by the lead author and last author for human studies investigating the effects of each antihypertensive on IL-6 levels. Specific search terms related to "anti-inflammation", "interleukins", "hypertension", and "blood pressure control" were used for each medication. Abstracts were initially collected and screened. Then, the full articles meeting the criteria were reviewed to determine if a direct IL-6 lowering effect was reported for the antihypertensive medication. Based on this evaluation, the antihypertensives were categorized as either possessing or lacking IL-6 lowering properties (Appendix 2; Supplementary Material). Notably, the literature search found evidence for IL-6 lowering properties in Angiotensin Converting Enzyme (ACE) inhibitors (ramipril), Angiotensin Receptor Blockers (ARBs) (valsartan), and some Calcium Channel Blockers (CCBs) (amlodipine and barnidipine) (Appendix 2; Supplementary Material).

Participants on long-term antihypertensive therapy often use a combination of medications, with occasional switching between drugs. Similarly, hypertensive monotherapy was rare in our cohort. Due to the complexity of capturing these switching patterns and analyzing each combination, while maintaining adequate statistical power, we simplified the analysis by focusing on the presence or absence of IL-6 lowering properties in antihypertensive medications. Participants who used an antihypertensive medication with IL-6 lowering properties at any point during the study (baseline and/or follow-up) were categorized as having used antihypertensive medication with IL-6 lowering properties. Conversely, participants who did not use an anti-hypertensive medication with IL-6 lowering properties were classified as not having used one.

#### 2.2.2 Other baseline measurements

The following measurements were also obtained at baseline: age, sex, educational level, occupation, tobacco smoking, alcohol consumption, physical activity, psychological stress, body mass index (BMI), and comorbidity with diabetes mellitus and chronic kidney diseases (CKD). A detailed description of these measurements is provided in Appendix 1 (Supplementary Material).

#### 2.2.3 Follow-up measurements

Blood pressure follow-up was recorded in a seated position using a Microlife WatchBP machine in mmHg. The mean of the first two measurements (out of three) was recorded. Blood pressure control was defined by WHO criteria (systolic blood pressure <140 mmHg and diastolic blood pressure <90 mmHg). To assess medication adherence, participants were asked to bring their medications to the follow-up visit, like the baseline assessment. Those who did not bring their medications or reported discontinuing their antihypertensives were excluded from the final analysis, potentially indicating non-adherence to treatment (proxy measure).

#### 2.2.4 Statistical analysis

We used RStudio version 4.2.1 for all statistical analyses. Normally distributed data were presented as mean ± standard deviation, while skewed data are presented as median (interquartile range). Categorical data were presented as frequency (percentage). Age and sex adjusted proportions of blood pressure control per antihypertensive class and IL-6 lowering properties were calculated with the Direct standardization package (total study population as the standard).

Logistic regression models were employed to assess the association between using antihypertensive medications with and without IL-6 lowering properties throughout the study and blood pressure control at follow-up. Initially, we performed analyses within medication classes (e.g., CCBs with vs. without IL-6 lowering properties) to minimize variations caused by class differences (inter-class heterogeneity). Subsequently, we conducted an inter-class comparison for medications showing significant intra-class effects.

We first adjusted for age and sex, and then subsequently for other potential confounders (education level, alcohol consumption, tobacco smoking, tobacco smoking, physical activity level, body mass index (BMI), psychosocial stress, presence of diabetes mellitus, and chronic kidney disease). Missing values were present for education, smoking, physical activity, alcohol consumption, and perceived stress, but in less than 2.5 % of the study sample. Due to this low missingness, missing values were not imputed. While acknowledging the potential influence of ethnicity on the relationship between medication use and blood pressure control, we opted to explore this factor in separate analyses rather than include it as a covariate in the primary models (see sensitivity analyses). Odds ratios (ORs) and their corresponding 95 % confidence intervals (CIs) are reported. All statistical tests were two-tailed with an alpha level of 0.05.

#### 2.2.5 Sensitivity analyses

To explore our hypothesis that the superiority of anti-hypertensives with IL6-lowering properties on blood pressure control would be more pronounced in ethnic minorities with higher inflammation levels compared to the Dutch group (known to have lower systemic inflammation) [8], we examined ethnic-specific associations in models stratified by ethnicity. Subgroup analyses excluded Moroccans (N = 39) and Turks (N = 29) due to limited sample sizes. We focused solely on the statistically significant associations identified in the intra-class analyses. Additionally, we compared the baseline characteristics between the antihypertensives with and without IL-6 lowering properties to further understand the findings from the initial population characteristics.

## 3 Results

### 3.1 Baseline characteristics

The study included participants with the following ethnic composition: 281 (18 %) were Dutch, 318 (21 %) were South-Asian Surinamese, 503 (33 %) were African Surinamese, 208 (14 %) were Ghanaian, 93 (6.2 %) were Turkish, and 107 (6.3 %) were Moroccan (Table 1). The median age was 57 years with an interquartile range (IQR) of 52–62 years. Among the participants, 62 % (N = 930) were female. Approximately 17 % (N = 254) of the participants reported being current smokers, and more than 78 % (N = 1163) reported a low level of alcohol consumption. A total of 29 % (N = 436) of the participants had diabetes mellitus, while 5 % (N = 70) had chronic kidney disease (CKD). The median score for BMI in the overall study population was 28.5, with an IQR of 25.8–32.0.

**Table 1:** Baseline characteristics of participants included in the study.

Characteristics	Total N = 1510	% or IQR
**Ethnicity, N(%)**		
Dutch	281	18.2
South Asian Surinamese	318	21.1
African Surinamese	508	33.3
Ghanain	208	13.8
Turkish	93	6.2
Moroccan	107	7.1
**Sex N(%)**		
Male	580	38.4
Female	930	61.6
**Age (years), median (IQR)**		
	57	52–62
**Level of Education, N (%)**		
Elementary	275	18.7
Primary	543	36.9
Secondary	354	24.0
Tertiary	300	20.4
**Smoking, N (%)**		
Yes	254	17.2
Never	836	56.5
Former	389	26.3
**Alcohol consumption, N (%)**^a^		
Low	1163	78.7
Moderate	236	16.0
High	79	5.3
**Norm for physical activity, N (%)**^b^		
Yes	969	65.1
No	520	34.9
**Perceived stress, N (%)**^c^		
Never	586	39.5
Some periods	616	41.5
Several periods	187	12.6
Permanent	94	6.3
**Diabetes Mellitus, N (%)**^d^		
Yes	436	29.0
No	1069	71.0
**Chronic kidney Disease (CKD), N (%)**^e^		
Yes	70	4.7
No	1434	95.3
**Body mass index (BMI), Median (IQR)**^f^		
	28.5	28.5–32.0
**Systolic BP in mmHg, Median (IQR)**^g^		
	137	127–148
**Diastolic BP in mmHg, Median (IQR)**^g^		
	81	78–88

∗Column totals do not always add to 100 % due to missing values. Missing values for education **N = 38**, Smoking **N = 31**, alcohol consumption **N =32** physical activity **N = 21**, perceived stress **N = 27**.

a low (men 0–4 glasses per week (gl/w), women 0–2 gl/w), moderate (men 5–14 gl/w, women 3–7 gl/w), and high (men >14 gl/w, women >7 gl/wk).

b Achieving the norm for physical activity is defined as ≥ 5 days/week 30 min moderately to high activity.

c This is defined as the combined stress score for work and home.

d Based on self-report or increased fasting glucose ( ≥ 7 mmol/l) or use of glucose lowering medication.

e CKD is defined as kidney failure (stage 3a and above) based on CKD-EPI 2021 eGFR, without adjustment for race (Inker et al., 2021).

f Body mass index presented in Kg/m.^2^.

g measured in seated position (mean of 2 measurements).

### 3.2 Antihypertensive medications and blood pressure control at follow up

**Fig. 2:**
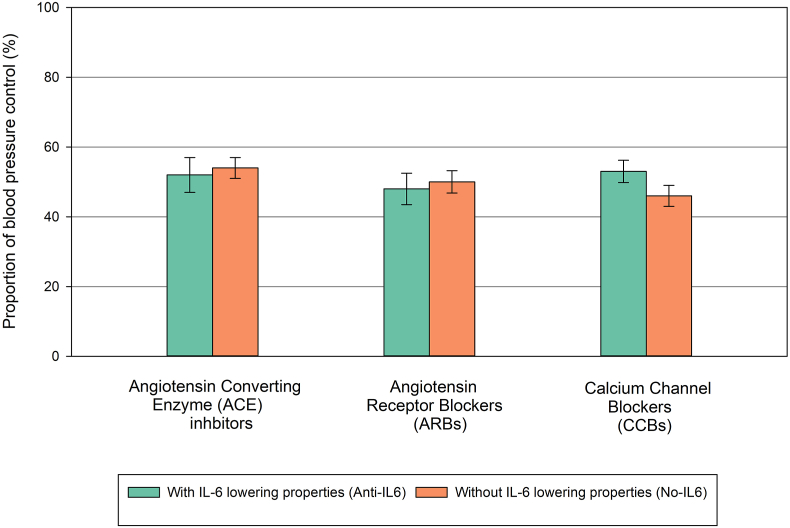
Age and sex adjusted proportions of blood pressure control across anti-hypertension classes. Total N = 1510, Dutch N = 281, South Asian Surinamese N = 318, African Surinamese N = 508, Ghanaian N = 208, Turkish N = 93, Moroccan N = 107.

CCBs were the most prescribed medication class, with 790 participants (52 %). This was followed by ACE inhibitors (447 participants, 30 %) and ARBs (445 participants, 30 %; Table 2). Overall, 54 % of the study population achieved blood pressure control at follow-up (N = 816). When comparing the age and sex-adjusted proportions of blood pressure control across medication classes: ACE inhibitors with IL-6 lowering properties (as reported in literature) showed a control proportion of 52 %, compared to 54 % in ACE inhibitors without IL-6 properties. ARBs, whether with or without IL-6 lowering properties, exhibited lower proportions, with 48 % and 50 % respectively. Among CCBs, those with IL-6 lowering properties had a control proportion of 54 %, whereas CCBs lacking IL-6 lowering properties showed a lower proportion of 45 % (Fig. 2).

**Table 2:** Medication use at some point in study the period.

Medication class	Total N = 1510	Percentage usage (%)
ACE inhibitors	447	29.6
ARB	445	29.5
Beta blockers	118	7.8
Calcium antagonists	790	52.3
Diuretics	46	3.0
Othersi	118	7.8

Total usage of the medication use at baseline and/or at follow up. Totals are over 100 % because medications were used in combination.

Others refers to patients using other classes of AHMs such as vasodilators, selective aldosterone antagonists, renin inhibitors, etc.

Within antihypertensive classes, the use of ACE-inhibitors with IL-6 lowering properties (ramipril) was not associated with better blood pressure control at follow-up compared to ACE-inhibitors without such properties (aOR 0.71, 95 % CI 0.40 to 1.18; Table 3). Similarly, the use of ARBs with IL-6 lowering properties (valsartan) was not associated with better blood pressure control at follow-up compared to ARBs without such properties (aOR 0.69, 95 % CI 0.44 to 1.06). In contrast, superior blood pressure control at follow-up was observed in participants treated with CCBs with IL-6 lowering properties (amlodipine and barnidipine), compared to those treated with CCBs without such properties (aOR 1.41, 95 % CI 1.05–1.90).

**Table 3:** Within-class comparison: Antihypertensives with and without IL6 lowering properties on blood pressure control.

	N	Model 1 - OR (95 % CI)a	Model 2 - OR (95 % CI)a	Model 3 - OR (95 % CI)a
Participants on ACE inhibitors (N = 447)				
Non-IL6b	361	1.00 (ref)	1.00 (ref)	1.00 (ref)
Anti-IL6b	86	0.75 [0.47–1.19]	0.75 [0.47–1.19]	0.71 [0.40–1.18]
Participants on Angiotensin receptor blockers (ARB's)(N = 385)				
Non-IL6	285	1.00 (ref)	1.00 (ref)	1.00 (ref)
Anti-IL6	160	0.89 [0.60–1.31]	0.87 [0.58–1.25]	0.69 [0.44–1.06]
Participants on Calcium channel blockers (N = 790)				
Non-IL6	356	1.00 (ref)	1.00 (ref)	1.00 (ref)
Anti-IL6	434	**1.37 [1.03**–**1.82]**	**1.37 [1.03**–**1.82]**	**1.41 [1.05**–**1.90]**

Odds ratios (ORs) and their corresponding 95 % confidence intervals (CIs) were estimated using logistic regression analysis.Predictor = antihypertensive category, outcome = Blood pressure control (yes for better control, no for less optimal control) Model 1: unadjusted Model 2: Model 1 adjusted for age and sex Model 3: Model 2 adjusted for level of education, smoking, alcohol consumption, physical activity, BMI psychosocial stress, presence of diabetes, and presence of chronic kidney disease.Complete case analysis used in **Model 3: N=445/447** for ACE inhibitors, **N=369/385** for Angiotensin receptor blockers and **N=764/790** for Calcium channel blockers.

**Anti-IL6** refers to antihypertensive medications with IL-6 lowering properties and **non-IL6** refers to antihypertensive medications without IL-6 lowering properties.Other drug classes outside the ones presented here do not have any drugs with anti-IL6 properties and hence not included in this analysis.

Since the intra-class comparisons found a statistically significant difference in blood pressure control between CCBs with and without IL-6 properties, but not within ARBs and ACE inhibitors we compared the CCBs with IL-6 lowering properties to other drug classes (ARBs and ACE inhibitors). We found no significant differences in blood pressure control between participants treated with CCBs with IL-6 lowering properties compared to participants treated with other non-IL-6 lowering antihypertensives (excluding CCBs without IL-6 lowering properties) (aOR 1.05, 95 % CI 0.88–1.54; Table 4). Similarly, there were no statistically significant differences in blood pressure control when we compared participants on IL-6 lowering CCBs to all other participants (including non-IL-6 CCBs) (aOR 0.99, 95 % CI 0.79–1.32). Lastly, when comparing all participants on IL-6 lowering medications (amlodipine, barnidipine, valsartan, ramipril) to all participants without IL-6 lowering medications, there were no statistically significant differences in blood pressure control (aOR 0.93, 95 % CI 0.74–1.16).

**Table 4:** Between-class comparison: Antihypertensives with and without IL6 lowering properties on blood pressure control.

	N	Model 1 - OR (95 % CI)^a^	Model 2 - OR (95 % CI)a	Model 3 - OR (95 % CI)^a^
CCBs anti-IL6 versus non-IL6 antihypertensives in other classes b				
Non-IL6^e^	551	1.00 (ref)	1.00 (ref)	1.00 (ref)
CCBs anti-IL6^e^	434	1.08 [0.84–1.38]	1.08 [0.84–1.39]	1.05 [0.88–1.54]
CCBs anti-IL6 versus all other antihypertensives c				
All other meds	1076	1.00 (ref)	1.00 (ref)	1.00 (ref)
CCBs anti-IL6	434	0.99 [0.79–1.24]	1.00 [0.80–1.25]	0.99 [0.79–1.32]
All anti-IL6 antihypertensives vs all non-IL6 antihypertensives d				
Non-IL6	907	1.00 (ref)	1.00 (ref)	1.00 (ref)
Anti-IL6	603	0.95 [0.77–1.17]	0.95 [0.77–1.17]	0.93 [0.74–1.16]

a Odds ratios (ORs) and their corresponding 95 % confidence intervals (CIs) were estimated using logistic regression analysis.Predictor = antihypertensive category, outcome = Blood pressure control (yes for better control, no for less optimal control). Model 1: unadjusted Model 2: Model 1 adjusted for age and sex Model 3: Model 2 adjusted for level of education, smoking, alcohol consumption, physical activity, BMI, psychosocial stress, diabetes, and presence of chronic kidney disease.

b **CCBs** stands for calcium channel blockers. This analysis compares the participants treated with CCBs that have anti-IL6 properties and the participants treated with antihypertensives that have no IL-6 lowering properties (non-IL6) in all other classes. Note that this analysis **excludes** non-IL6 CCBs. Complete case analysis for model 3 N = 959/989.

c This analysis compares the participants treated with CCBs that have anti-IL6 properties and the participants treated with all other antihypertensives that have no IL-6 lowering properties. Note that this analysis **includes** non-IL6 CCBs. Complete case analysis for **model 3 N = 1472/1510**.

d This analysis compares all participants treated with anti-IL6 antihypertensives from all classes and participants treated with non-IL6 antihypertensives from all classes. Complete case analysis for model 3 N = 1472/1510.

e **Anti-IL6** refers to antihypertensive medications with IL-6 lowering properties and **non-IL6** refers to antihypertensive medications without IL-6 lowering properties.

### 3.3 Sensitivity analyses

We first compared the baseline characteristics of participants on CCBs with IL-6 lowering properties to those on CCBs without such properties to better understand initial characteristics. We found that baseline characteristics were generally similar between the two CCB groups, including the mean systolic blood pressure at baseline (143.59 mmHg; SD17.82) in the IL-6 lowering group compared to 144.24 mmHg (SD18.20) in the non-IL-6 lowering group) (Appendix 3).

**Fig. 3:**
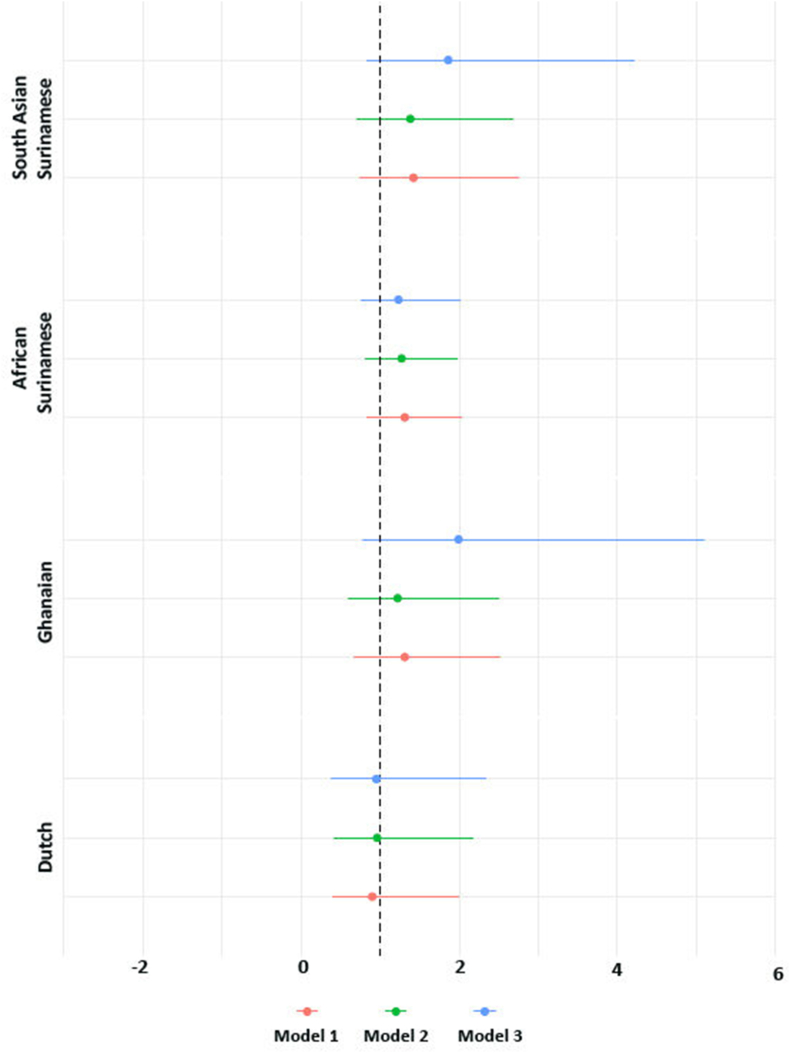
Stratified analyses on Calcium Channel Blockers with and without IL6 lowering properties by ethnicity. 51 out of 97 (53 %) on CCBs with IL6 lowering properties for Dutch, 88/151 (58 %) for South Asian Surinamese, 180/328 (55 %) for African Surinamese, and 72/146 (50 %) for Ghanaians. Model 1: unadjusted; Model 2: Model 1 adjusted for age and sex; Model 3: Model 2 adjusted for level of education, smoking, alcohol consumption, physical activity, BMI, psychosocial stress, diabetes, and presence of chronic kidney disease.

We stratified our analyses by ethnicity to understand how differences in blood pressure control between CCBs with and without IL-6-lowering properties in literature fared in populations with higher inflammatory rates (ethnic minorities) compared to the lower inflammatory rate group (Dutch). Although the findings did not reach statistical significance due to modest sample sizes (N = 97 for Dutch N = 151 for South Asian Surinamese, N = 328 for African Surinamese, and N = 146 for Ghanaians on CCBs), there was a notable trend. This trend indicated better blood pressure control during follow-up in ethnic minority populations treated with CCBs with IL-6-lowering properties compared to those treated with medications without such properties. Specifically, this trend appeared more prominent in Ghanaians (aOR 1.99, 95 % CI 0.78–5.10), South-Asian Surinamese (aOR 1.86, 95 % CI 0.83–4.20), and African Surinamese (aOR 1.23, 95 % CI 0.76–2.00) compared to the Dutch population (aOR 0.95, 95 % CI 0.39–2.33; Fig. 3).

## 4 Discussion

### 4.1 Key findings

Our study examined associations between the use of antihypertensive medications with and without IL-6 lowering properties (as reported in literature) and long-term blood pressure control in a multi-ancestry population in the Netherlands. Our findings suggest that amlodipine and barnidipine have superior blood pressure control compared to other CCBs (lercanidipine, nifedipine, verapamil, clevidipine, diltiazem). No significant association between IL-6 lowering properties in literature and blood pressure control was observed for other classes of antihypertensives, or for ethnic groups.

### 4.2 Discussion of key findings

We observed superior blood pressure control for amlodipine and barnidipine compared to those other CCBs (lercanidipine, nifedipine, verapamil, clevidipine, diltiazem). These two anti-hypertensives were grouped together based on their IL-6 Lowering properties in literature. As IL6-levels were not directly measured, the explanations for this finding might be IL6-related or also due to other unmeasured factors common to the two drugs.

With respect to IL-6 mechanisms, a possible explanation of this observation could be synergy in reducing vascular resistance and inflammation. Vascular resistance, defined as the impedance to blood flow within arterial vessels [13,14], and vascular inflammation, typified by endothelial dysfunction and inflammatory cascades [15], jointly contribute to the pathogenesis of hypertension [[13], [14], [15]]. Increased resistance to blood flow due to vasoconstriction and structural changes in blood vessel walls elevates pressure within the vessels, placing greater strain on the heart [14]. Concurrently, inflammatory mediators exacerbate endothelial dysfunction, further promoting vasoconstriction and vascular remodeling [15]. This interplay creates a vicious cycle, perpetuating hypertension and increasing the risk of cardiovascular complications [14,15]. Amlodipine is a dihydropyridine CCB that works by inhibiting calcium influx into smooth muscle cells via the L-type calcium channels, leading to vasodilation (lowering vascular resistance) and lower blood pressure [16]. Barnidipine is also a dihydropyridine CCB that blocks calcium channels in vascular smooth muscle cells, resulting in vasodilation and reduced peripheral resistance [17]. CCBs, such as amlodipine and barnidipine, have also been shown to modulate IL-6 signaling with a significant decrease of TNF-α and IL-6, lowering vascular inflammation [18,19]. The anti-inflammatory properties of these CCBs in addition to the vasodilatory effects could explain why CCBs with IL-6 lowering properties have superior blood pressure control than CCBs that work only on vasodilation.

With respect to unmeasured factors, the superior blood pressure control observed with amlodipine and barnidipine compared to other CCBs (lercanidipine, nifedipine, verapamil, clevidipine, diltiazem) may be partly attributed to differences in their pharmacodynamics, pharmacokinetics, and potential drug interactions. Amlodipine and barnidipine are long-acting dihydropyridine CCBs with extended half-lives (30–50 h for amlodipine and 20 h for barnidipine) [16,17], ensuring sustained calcium channel blockade and stable blood pressure control throughout the dosing interval. In contrast, shorter-acting CCBs like nifedipine and clevidipine require more frequent dosing or are primarily used in acute settings [20,21], potentially leading to less consistent blood pressure control in chronic management. Furthermore, amlodipine and barnidipine exhibit high vascular selectivity [22], targeting vascular smooth muscle with minimal cardiac effects, unlike non-dihydropyridine CCBs like verapamil and diltiazem [22], which act on both vascular and cardiac calcium channels and are often prescribed for arrhythmias or angina rather than hypertension alone [23,24]. Their lipophilicity also enhances tissue penetration, optimizing vasodilation and antihypertensive effects [16,17]. Additionally, drug interactions may play a role, as amlodipine and barnidipine, metabolized via the cytochrome P450 3A4 pathway, may interact with co-prescribed drugs like statins, potentially enhancing their antihypertensive efficacy [16,17]. These properties collectively distinguish amlodipine and barnidipine as more effective options for chronic blood pressure management compared to other CCBs in this context.

Additionally, across ethnic groups, we did not find significant associations between amlodipine and barnidipine and blood pressure control compared with other CCBs (lercanidipine, nifedipine, verapamil, clevidipine, diltiazem). However, there was a trend for superior effects in the ethnic minority groups compared to the Dutch. Without significant effects, the evidence was not conclusive. This lack of association could be due to smaller sample sizes resulting from splitting the dataset into smaller ethnic batches. Further studies should explore ethnicity with a larger sample size.

On the other hand, the lack of significant differences observed in other groups of antihypertensives with IL-6-lowering properties, such as ramipril and valsartan, may relate to differences in their underlying mechanisms of action, potency of IL-6 modulation, or other unmeasured pharmacokinetic, pharmacodynamic, or drug interaction effect. Ramipril is an angiotensin-converting enzyme (ACE) inhibitor [25]. It works by inhibiting the activity of the ACE enzyme produced in the lungs and kidneys, which plays a role in the production of angiotensin II, a potent vasoconstrictor [25]. By blocking ACE, Ramipril reduces the conversion of angiotensin I to angiotensin II, leading to arterial dilation, decreased peripheral resistance, and ultimately lowering blood pressure [25]. Valsartan is an angiotensin II receptor blocker (ARB) that works by selectively blocking the angiotensin II type 1 (AT1) receptors in various tissues, including endothelial cells [26]. Angiotensin II is a hormone that binds to these receptors and causes blood vessels to constrict, leading to an increase in blood pressure [26]. By blocking AT1 receptors, valsartan prevents the vasoconstrictive effects of angiotensin II. This leads to vasodilation, reduced peripheral resistance, and ultimately lowers BP [26].

Unmeasured factors such as pharmacokinetic properties (e.g., bioavailability, tissue penetration) and pharmacodynamic variability (e.g., receptor affinity or downstream signaling modulation) may also explain the lack of observed differences [26,27]. Additionally, potential drug-drug interactions, including concurrent medications metabolized by shared enzymatic pathways [28], could attenuate or enhance the efficacy of these drugs in lowering blood pressure and inflammation. For example, concurrent use of medications that induce or inhibit the cytochrome P450 system, which is involved in the metabolism of many antihypertensive drugs, might alter the effective concentrations of ramipril or valsartan, impacting their clinical outcomes [29]. These complexities highlight the multifactorial nature of drug effects and underscore the need for further research into the interplay between their pharmacological actions and inflammatory modulation [29].

This study provides important insights with potential implications for both clinical practice and scientific research. It identifies amlodipine and barnidipine as superior to other CCBs in controlling blood pressure, a novel finding that could influence the selection of CCBs in hypertension treatment in the future. However, the mechanisms underlying this observed superiority remain unclear. Investigating these two CCBs in comparison to others could uncover novel mechanisms of action, advancing our understanding of CCBs and their role in hypertension management. As such, this study serves as a valuable stepping stone toward personalized selection of CCBs for treatment. Further studies are needed to dissect the underlying mechanisms, including direct measurement of IL-6, as well as disentangling drug interactions, pharmacokinetics, and pharmacodynamics.

### 4.3 Strengths and limitations

Our study possesses notable strengths that contribute to its scientific rigor. Firstly, it incorporates a multi-ancestry population, encompassing diverse ethnic backgrounds. This enhances the generalizability of the findings and facilitates the exploration of potential health disparities within society. Secondly, the comprehensive evaluation by literature review of various antihypertensive medication classes helped classify us medication with documented effects in humans.

However, several limitations should be acknowledged. The inability to independently confirm the presence or absence of IL-6 lowering properties negatively affects the study's accuracy. While well documented in literature, the IL-6 effects might vary based on individual characteristics. Additionally, medication classification was based on existing literature, and antihypertensives not reported with respect to inflammation were categorized as lacking IL-6 lowering properties. This may not capture all potential IL-6 interactions accurately. Furthermore, we could not control all known and unknown confounders. Therefore, randomized controlled trials (RCTs) that control confounders are needed to definitively prove our hypothesis.

Not adjusting for multiple medication therapy is another limitation. Hypertensive monotherapy was rare in our cohort; hence we could not explore the associations in participants who sorely used one medication. Participants were also free to switch drugs during the study, which could affect the duration of medication use and its potential effect. Unfortunately, we did not perform a separate analysis to measure the degree of drug switching between baseline and follow-up. Additionally, in a two-time-timepoint study like ours (baseline and follow-up), pinpointing the exact timing of drug switches can be challenging.

## 5 Conclusion

Amlodipine and barnidipine were associated with better BP control compared to other CCBs; however, the underlying mechanisms remain unclear. Further research is needed to directly measure IL-6 levels and investigate whether inflammatory pathways contribute to these observed differences in BP control.

## CRediT authorship contribution statement

**Hillman Batuo:** Writing – review & editing, Writing – original draft, Methodology, Investigation, Formal analysis, Data curation, Conceptualization. **Eva van der Linden:** Writing – review & editing, Supervision. **Henrike Galenkamp:** Writing – review & editing, Methodology. **Eric Moll van Charante:** Writing – review & editing, Supervision. **Bert-Jan van der Born:** Writing – review & editing, Supervision. **Felix P. Chilunga:** Writing – review & editing, Writing – original draft, Validation, Supervision, Investigation, Formal analysis, Data curation.

## Data sharing statement

The HELIUS data are owned by the Amsterdam University Medical Centers, location AMC in Amsterdam, The Netherlands. Any researcher can request the data by submitting a proposal to the HELIUS Executive Board as outlined at http://www.heliusstudy.nl/en/researchers/collaboration, by email: heliuscoordinator@amsterdamumc.nl. The HELIUS Executive Board will check proposals for compatibility with the general objectives, ethical approvals, and informed consent forms of the HELIUS study. There are no other restrictions to obtaining the data and all data requests will be processed in the same manner.

## Funding

The HELIUS study is funded by the Dutch Heart Foundation (grant number 2010T084), The Netherlands Organization for Health Research and Development (10.13039/501100001826ZonMw grant number 200500003), and the European Commission (grant number 278901).

## Competing interests

The author declares that there is no conflict of interest.
