# Supplementary material for: Effects of antihypertensives with and without IL-6 lowering properties on long-term blood pressure control: The prospective HELIUS cohort
Source: Int J Cardiol Cardiovasc Risk Prev. 2024 Dec 11;24:200358. doi: 10.1016/j.ijcrp.2024.200358 (PMC11699610; doi:10.1016/j.ijcrp.2024.200358)
Supplement: Multimedia component 1 [file mmc1.pdf]

Appendix 1: Measurements at baseline in detail

For ethnicity, a person was defined as of non-Dutch ethnic origin if he/she fulfilled one of two criteria: (1) he/she was born outside the Netherlands, and has at least one parent born outside the Netherlands (first generation) or (2) he/she was born in the Netherlands but both parents were born outside the Netherlands (second generation). For the Dutch sample, we invited people who were born in the Netherlands and whose parents were born in the Netherlands. A limitation of the country of birth indicator for ethnicity is that people who are born in the same country might have a different ethnic background, which in the Dutch context is applicable to the Surinamese population. Therefore, after data collection, participants of Surinamese ethnic origin were further classified according to self-reported ethnic origin (obtained by questionnaire) into 'African', 'South-Asian', 'Javanese', or 'other'.[1]

Age was recorded in years, while sex was recorded sex as males and females. Educational level was classified into four groups, based on the highest qualification gained either in the Netherlands or in the country of origin; 1. Never been to school or elementary schooling, 2. lower vocational schooling or lower secondary schooling, 3. intermediate vocational schooling or intermediate/higher secondary education schooling, 4. higher vocational schooling or university. Occupational level was classified according to the Dutch Standard Occupational Classification system, which provides an extensive systematic list of all professions in the Dutch system.[2] Occupational level was categorised into elementary, lower, medium, higher, and scientific. Smoking status was assessed by participants' current smoking status. Use of alcohol was based on the reported units of alcohol intake per week and categorized into low (men 0-4 glasses/week and women 0-2 glasses/week), moderate (men 5-14 glasses/week and women 3-7 glasses/week), or high (men >14 glasses/week, women >7 glasses/week).[3] Physical activity was assessed using the Short QUestionnaire to Assess Health enhancing physical activity (SQUASH) and was dichotomized into ≥5 days, 30 min/day versus <5 days, 30 min/day.[4]

Psychological stress resulting from situations experienced at home or at work in the past 12 months was measured by the psychological stress scale created by the INTERHEART study.[5] Participants were asked about the prevalence of stress at home or at work and could answer: 'never/does not apply' = 1, 'some periods' = 2, 'several periods' = 3 or 'constantly' = 4. Weight was measured without shoes and in light clothing with SECA 877 scales to the nearest 0.1 kg. Body mass index (BMI) was determined by dividing measured body weight (kg) by height squared (m^2).

During the physical examination a fasting venous blood sample and morning urine sample were obtained. This was used to assess the estimated Glomerular Filtration Rate (eGFR) and the Albumin to Creatinine Ratio (ACR). Fasting glucose was measured by spectrophotometry, using hexokinase as primary enzyme (Roche Diagnostics, Japan). Diabetes mellitus was defined by fasting plasma glucose concentration of

≥7.0 mmol/l and/or based on self-report and/or the use of glucose-lowering agents per WHO criteria. eGFR was measured via serum creatinine concentration (in µmol/L) which was determined by an enzymatic method (Roche C702 at the C8000 platform). Chronic Kidney disease (CKD) was defined based on CKD-EPI 2021 eGFR equation (stage 3a and above), without adjustment for race.[6]

REFERENCES

1. Stronks K, Kulu-Glasgow I, Agyemang C. The utility of 'country of birth'for the classification of ethnic groups in health research: the Dutch experience. *Ethnicity & health* 2009; **14**(3): 255-69.

2. Netherlands S. Dutch Standard Classification of Occupations (SBC), 1992. *The Hague: Statistics Netherlands* 1992.

3. Dufour MC. What is moderate drinking?: Defining "drinks" and drinking levels. *Alcohol Research & Health* 1999; **23**(1): 5.

4. Wagenmakers R, Akker-Scheek Ivd, Groothoff JW, et al. Reliability and validity of the short questionnaire to assess health-enhancing physical activity (SQUASH) in patients after total hip arthroplasty. *BMC Musculoskeletal Disorders* 2008; **9**: 1-9.

5. Rosengren A, Hawken S, Ôunpuu S, et al. Association of psychosocial risk factors with risk of acute myocardial infarction in 11 119 cases and 13 648 controls from 52 countries (the INTERHEART study): case-control study. *The Lancet* 2004; **364**(9438): 953-62.

6. Hundemer GL, White CA, Norman PA, et al. Performance of the 2021 Race-Free CKD-EPI Creatinine-and Cystatin C–Based Estimated GFR Equations Among Kidney Transplant Recipients. *American Journal of Kidney Diseases* 2022; **80**(4): 462-72. e1.

Appendix 2. PRISMA 2020 flow diagram for systematic reviews

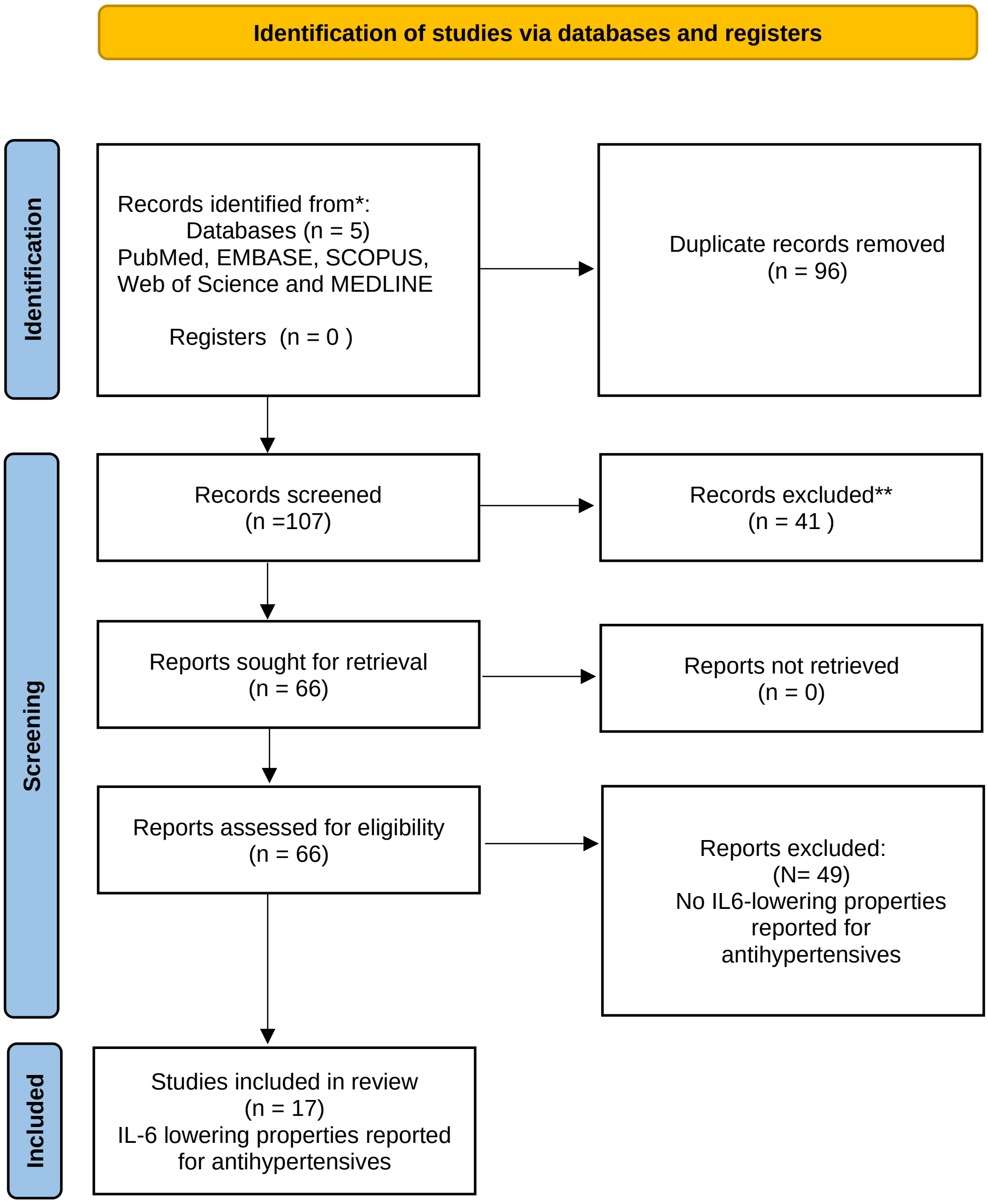

Source: Page MJ, et al. BMJ 2021;372:n71. doi: 10.1136/bmj.n71.

This work is licensed under CC BY 4.0. To view a copy of this license, visit https://creativecommons.org/licenses/by/4.0/

Appendix 2: Classification of anti-hypertensives into anti-IL-6 versus no-IL-6 based on a systematic review of literature.

Medication (ATC codes)	Anti-inflammatory properties	Mechanism of Action (reference article)	IL-6 lowering properties
**ACE blockers**			
Enalapril (C09AA02)	No		No
Lisinopril (C09AA03)	No		No
Perindopril (C09AA04)	Yes	Weak increase in IL-10 [2] and Increase level of Thrombospondin -1 (TSP-1) [3]	No
Ramipril (C09AA05)	Yes	Reduces level of hs-CRP, IL-6 and TNF-alpha [4]	Yes
Captopril (C09AA01)	Yes	Inactivation of NF-kB signaling [5]	No
Zofenopril (Z09AA15)	No		No
Fosinopril (C09AA09)	No		No
Quinapril (C09AA06)	Yes	Suppression of inflammatory arthritis, reduction in expression of articular expression of TNF-alpha [6]	No
**Beta blockers; beta-1 selective**			
Atenolol (C07AB03)	No		No
Bisoprolol (C07AB07)	Yes	Reduction in hsCRP levels[7]	No
Metoprolol (C07AB02)	No		No
Nebivolol (C07AB12)	Yes	Downregulation of interleukin-1 alpha, cyclooxegyenase-2, tumor-necrosis factor (TNF), PDGF-A. [8]	No
Propranolol (C07AA05)	No		No
Timolol (S01ED01)	No		No
**Beta-blockers; Alpha activity**			
Labetalol (C07AG01)	No		No
Carvedilol (C07AG02)	No		No
**Beta-blockers; intrinsic sympathomimetic**			
Acebutolol (C07AB04)	No		No
Pindolol (C07AA03)	No		No
**Angiotensin receptor blockers (ARB's)**			

Candesartan (C09CA06)	Yes	Significantly diminishes the response to LPS, including reduction of pro-inflammatory cytokine release to the general circulation and decreased production and release of the pro-inflammatory adrenal hormone aldosterone [9]	No
Losartan (C09CA01)	Yes	Acting as immunomodulator preventing development of CD14+ CD16+ pro-inflammatory monocytes in hemodialysis patients [10]	No
Telmisartan (C09CA07)	Yes	From studies with cultured human umbilical vein endothelial cell (HUVEC's) – Reduced the TNF-alpha stimulated VCAM-1 in a concentration dependent manner [11]	No
Valsartan (C09CA03)	Yes	Mention worthy but not significant decrease in hsCRP and VCAM-1 Significant decrease in serum TNF-alpha and IL-6. [12]	Yes
Olmesartan (C09CA08)	Yes	Significantly diminishes the response to LPS, including reduction of pro-inflammatory cytokine release to the general circulation and decreased production and release of the pro-inflammatory adrenal hormone aldosterone [9]	No
Eprosartan (C09CA02)	Yes	Significantly diminishes the response to LPS, including reduction of pro-inflammatory cytokine release to the general circulation and decreased production and release of the pro-inflammatory adrenal hormone aldosterone [9]	No
Azilsartan (C09CA09)	Yes	Significantly diminishes the response to LPS, including reduction of pro-inflammatory cytokine release to the general circulation and decreased production and release of the pro-inflammatory adrenal hormone aldosterone [9]	No
Irbesartan (C09CA04)	Yes	Significantly diminishes the response to LPS, including reduction of pro-inflammatory cytokine release to the general circulation and decreased production and release of the pro-inflammatory adrenal hormone aldosterone [9]	No
**Vasodilators**			

Hydralazine (C02DB02)	No		No
Minoxidil (C02DC01)	No		No
**Calcium antagonists**			
Amlodipine (C08CA01)	Yes	Lowers plasma IL-6 levels [1]	Yes
Lercanidipine (C08CA13)	Yes	Significant reduction in circulating levels of IL-18, MCP-1, sICAm-1, and CRP [13]	No
Lacidipine (C08CA09)	No		No
Nifedipine (C08CA05)	No		No
Verapamil (C08DA01)	Yes	Decrease in MMP-2/MMP-9 (Matrix metalloproteinases) activity in human PBMCs [14]	No
Clevidipine (C08CA16)	No		No
Diltiazem (C08DB01)	No		No
Barnidipine (C08CA12)	Yes	Reduction in TNF-alpha, IL-6, and Hs-CRP levels.[15]	Yes
**Selective Aldosterone antagonists**			
Eplerenone (C03DA04)	No		No
Spironolactone (C03DA01)	No		No
**Central-acting alpha 2-agonists**			
Methyldopa (C02AB01)	No		No
Clonidine (C02AC01)	No		No
Guanfacine (C02AC02)	No		No
**Thiazide Diuretics**			
Chlorthalidone (C03BA04)	No		No
Hydrochlorothiazide (C03AA03)	No		No
Metolazone (C03BA08)	No		No
Indapamide (C03BA11)	No		No
**Potassium sparing diuretics**			
Triamterene (C03DB02)	No		No
Amiloride (C03DB01)	No		No
**Loop diuretics**			
Furosemide (C03CA01)	No		No
Torasemide/Torsemide (C03CA04)	No		No
Bumetanide (C03CA02)	No		No
**Renin inhibitors/combos**			
Aliskiren (C09XA02)	Yes	Decrease in MCP-1 concentration [16]	No

**Antihypertensives Alpha-blockers**			
Prazosin (C02CA01)	No		No
Terazosin (G04CA03)	No		No
Doxazosin (C02CA04)	Yes	Decrease in hsCRP [17]	No
**Other antihypertensives**			
Reserpine (C02AA02)	No		No
Sotalol (C07AA07)	No		No

We conducted a systematic search of literature for each of the 56 antihypertensive medications used in the HELIUS cohort. Prominent databases like PubMed, EMBASE, SCOPUS, Web of Science and MEDLINE were searched for human studies investigating the effects of antihypertensives on IL-6 levels. Specific search terms related to "anti-inflammation," "interleukins," "hypertension," and "blood pressure control" were used for each medication. Abstracts were initially collected and screened. Then, the full articles meeting the criteria were reviewed to determine if a direct IL-6 lowering effect was reported for the antihypertensive medication. Based on this evaluation, the antihypertensives were categorized as either possessing or lacking IL-6 lowering properties

REFERENCES:

1. Mohler ER, Sorensen LC, Ghali JK, et al. Role of cytokines in the mechanism of action of amlodipine: the PRAISE heart failure trial. *Journal of the American College of Cardiology* 1997; **30**(1): 35-41.

2. Gilowski W, Krysiak R, Marek B, Okopień B. The effect of short-term perindopril and telmisartan treatment on circulating levels of anti-inflammatory cytokines in hypertensive patients. *Endokrynologia Polska* 2018; **69**(6): 667-74.

3. Buda V, Andor M, Petrescu L, et al. Perindopril induces TSP-1 expression in hypertensive patients with endothelial dysfunction in chronic treatment. *International Journal of Molecular Sciences* 2017; **18**(2): 348.

4. Ateya AM, El Hakim I, Shahin SM, El Borolossy R, Kreutz R, Sabri NA. Effects of ramipril on biomarkers of endothelial dysfunction and inflammation in hypertensive children on maintenance hemodialysis: the SEARCH randomized placebo-controlled trial. *Hypertension* 2022; **79**(8): 1856-65.

5. Gan Z, Huang D, Jiang J, Li Y, Li H, Ke Y. Captopril alleviates hypertension-induced renal damage, inflammation, and NF-κB activation. *Brazilian Journal of Medical and Biological Research* 2018; **51**: e7338.

6. Dalbeth N, Edwards J, Fairchild S, Callan M, Hall F. The non-thiol angiotensin-converting enzyme inhibitor quinapril suppresses inflammatory arthritis. *Rheumatology* 2005; **44**(1): 24-31.

7. Toyoda S, Haruyama A, Inami S, et al. Effects of carvedilol vs bisoprolol on inflammation and oxidative stress in patients with chronic heart failure. *Journal of cardiology* 2020; **75**(2): 140-7.

8. Wolf SC, Sauter G, Jobst J, Kempf VA, Risler T, Brehm BR. Major differences in gene expression in human coronary smooth muscle cells after nebivolol or metoprolol treatment. *International journal of cardiology* 2008; **125**(1): 4-10.

9. Benicky J, Sánchez-Lemus E, Pavel J, Saavedra JM. Anti-inflammatory effects of angiotensin receptor blockers in the brain and the periphery. *Cellular and molecular neurobiology* 2009; **29**: 781-92.

10. Merino A, Alvarez-Lara MA, Ramirez R, Carracedo J, Martin-Malo A, Aljama P. Losartan prevents the development of the pro-inflammatory monocytes CD14+ CD16+ in haemodialysis patients. *Nephrology Dialysis Transplantation* 2012; **27**(7): 2907-12.

11. Cianchetti S, Del Fiorentino A, Colognato R, Di Stefano R, Franzoni F, Pedrinelli R. Anti-inflammatory and anti-oxidant properties of telmisartan in cultured human umbilical vein endothelial cells. *Atherosclerosis* 2008; **198**(1): 22-8.

12. Janic M, Lunder M, Prezelj M, Šabovic M. A combination of low-dose fluvastatin and valsartan decreases inflammation and oxidative stress in apparently healthy middle-aged males. *Journal of cardiopulmonary rehabilitation and prevention* 2014; **34**(3): 208-12.

13. De Ciuceis C, Rossini C, Tincani A, et al. Effect of antihypertensive treatment with lercanidipine on endothelial progenitor cells and inflammation in patients with mild to moderate essential hypertension. *Blood Pressure* 2016; **25**(6): 337-43.

14. Hajighasemi F, Kakadezfuli N. Suppression of gelatinase activity in human peripheral blood mononuclear cells by verapamil. *Cell Journal (Yakhteh)* 2014; **16**(1): 11.

15. Derosa G, Mugellini A, Pesce RM, D'Angelo A, Maffioli P. Perindopril and barnidipine alone or combined with simvastatin on hepatic steatosis and inflammatory parameters in hypertensive patients. *European Journal of Pharmacology* 2015; **766**: 31-6.

16. Makówka A, Olejniczak-Fortak M, Nowicki M. A comparison of the antihypertensive and anti-inflammatory effects of aliskiren and ramipril add-on therapy in peritoneal dialysis patients–a pilot open label study. *Kidney and Blood Pressure Research* 2013; **36**(1): 18-25.

17. Derosa G, Cicero AF, D'Angelo A, et al. Effect of doxazosin on C-reactive protein plasma levels and on nitric oxide in patients with hypertension. *Journal of cardiovascular pharmacology* 2006; **47**(4): 508-12.

Appendix 3. Characteristics of participants on calcium channel blockers with and without IL-6 lowering properties

**Characteristic**	**Calcium Channel Blockers**		
	**Without IL-6 lowering Properties (No-IL6) N= 356**	**With IL-6 lowering properties (Anti-IL6) N=434**	**P-value for diffrence between groups**
**Baseline characteristics**			
**Ethnicity, N (%)**			**0.421**
Dutch	46 (12.9)	51 (11.8)	
South Asian Surinamese	63 (17.7)	88 (20.3)	
African Surinamese	148 (41.6)	180 (41.5)	
Ghanain	74 (20.8)	72 (16.6)	
Turkish	10 (2.8)	19 (4.4)	
Moroccan	15 (4.2)	24 (5.5)	
**Sex, N(%)**			
Male	144 (40.4)	162 (37.3)	
Female	212 (59.6)	272 (62.7)	
**Age (years), mean (SD)**			**0.811**
	56.49 (7.89)	56.67 (6.76)	
**Level of Education N (%)**			**0.114**
Elementary	56 (16.4)	88 (20.9)	
Primary	150 (43.9)	153 (36.3)	
Secondary	74 (21.6)	108 (25.6)	
Tertiary	62 (18.1)	73 (17.3)	
**Smoking, N (%)**			**0.783**
Yes	68 (19.5)	74 (17.5)	
Never	194 (55.7)	247 (58.4)	
Former	86 (24.7)	102 (24.1)	
**Alcohol consumption, N (%) [a]**			**0.876**
Low	283 (81.6)	343 (81.1)	
Moderate	50 (14.4)	59 (13.9)	
High	14 (4.0)	21 (5.0)	
**Norm for physical activity, N (%) [b]**			**0.345**
Yes	228 (65.1)	262 (61.4)	
No	128 (34.9)	172 (38.6)	
**Perceived stress N, (%) [c]**			**0.622**
Never	148 (42.7)	175 (41.0)	
Some periods	140 (40.3)	162 (37.9)	
Several periods	39 (11.2)	61 (14.3)	
Permanent	20 (5.8)	29 (6.8)	
**Diabetes, N (%) [d]**			**0.357**
Yes	100 (28.2)	136 (31.4)	
No	334 (76.8)	298 (68.6)	
**Chronic kidney Disease (CKD) N (%) [e]**			**0.012**
Yes	30 (8.5)	18 (4.2)	
No	326 (91.4)	416 (95.8)	
**Body mass index (BMI), Mean (SD) [f]**			**0.391**
	29.10 (4.77)	29.60 (4.98)	
**Systolic BP in mmHg, Mean (SD) [g]**			**0.125**
	144.24 (18.20)	143.59 (17.82)	
**Diastolic BP in mmHg, Mean (SD) [g]**			**0.114**
	86.78 (10.69)	85.44 (9.97)	
**Follow up blood pressure measurements (6 years later)**			
**Systolic BP in mmHg, Mean (SD)**			**0.017**
	142.46 (19.79)	138.78 (16.17)	
**Diastolic BP in mmHg, Mean (SD)**			**0.114**

	82.84 (10.61)	81.55 (9.32)	

**Column totals do not always add to 100% due to missing values.*

[a] *low (men 0-4 glasses per week (gl/w), women 0-2 gl/w), moderate (men 5-14 gl/w, women 3-7 gl/w), and high (men >14 gl/w, women >7 gl/wk).*

[b] *Achieving the norm for physical activity is defined as >=5 days/week 30 min moderately to high activity*

[c] *This is defined as the combined stress score for work and home.*

[d] *Based on self-report or increased fasting glucose (>=7 mmol/l) or use of glucose lowering medication*

[e] *CKD is defined as kidney failure (stage 3a and above) based on CKD-EPI 2021 eGFR, without adjustment for race (Inker et al, 2021)*

[f] *Body mass index presented in Kg/m^2*

[g] *measured in seated position (mean of 2 measurements)*

[h] *BP control is defined as a systolic BP (measure in seated position) of less than 140mmHg AND a diastolic BP of less than 90mmHg.*

[i] *Others refers to patients using other classes of AHMs such as vasodilators, selective aldosterone antagonists, renin inhibitors, etc.*
